# Reduced acquisition times in whole body bone scintigraphy using a noise-reducing Pixon®-algorithm—a qualitative evaluation study

**DOI:** 10.1186/s13550-015-0127-x

**Published:** 2015-09-16

**Authors:** Oscar Ardenfors, Ulrika Svanholm, Hans Jacobsson, Patricia Sandqvist, Per Grybäck, Cathrine Jonsson

**Affiliations:** Department of Medical Physics, Karolinska University Hospital, Stockholm, 17176 Sweden; Department of Radiology, Karolinska University Hospital, Stockholm, 17176 Sweden

**Keywords:** Pixon-algorithm, Half-time scanning, Planar imaging, Whole body bone scintigraphy, Visual grading

## Abstract

**Background:**

Reducing scan-time while maintaining sufficient image quality is a common issue in nuclear medicine diagnostics. This matter can be addressed by different post-processing methods such as Pixon® image processing. The aim of the present study was to evaluate if a commercially available noise-reducing Pixon-algorithm applied on whole body bone scintigraphy acquired with half the standard scan-time could provide the same clinical information as full scan-time non-processed images.

**Methods:**

Twenty patients were administered with 500 MBq ^99m^Tc-diphosphonate and scanned on a Siemens Symbia T16 system. Each patient was first imaged using a standard clinical protocol and subsequently imaged using a protocol with half the standard scan-time. Half-time images were processed using a commercially available software package, Enhanced Planar Processing, from Siemens. All images were anonymized and visually evaluated with regard to clinically relevant lesion detectability by three experienced nuclear medicine physicians. The result of this evaluation was grouped into four BMI intervals to investigate the performance of the algorithm with regard to different patient size. Also, a comparison study was performed where the physicians compared the standard image and the processed half-time image corresponding to the same patient with regard to lesion detectability, image noise, and artifacts.

**Results:**

The results showed that 93 % of the processed half-time images and 98 % of the standard images were rated as sufficient or good with regard to lesion detectability. The processed half-time images were predominately considered sufficient (65 %), whereas the majority of the standard images were graded as good (83 %). The performance of the algorithm was unaffected by patient size as the average grading of all half-time processed images was constant independent of patient BMI. The comparison study showed that the standard images were rated superior with regard to lesion detectability, image noise, and artifacts, in 32, 65, and 23 % of the evaluations, respectively.

**Conclusions:**

The results indicate that the Pixon Enhanced Planar Processing does not fully compensate for the loss of counts associated with reducing the scan-time in half for whole body bone scintigraphies. The findings showed that implementing the Pixon-algorithm on images acquired with half the acquisition time in overall provide sufficient clinical information regardless of patient size. The half-time processed images were predominantly graded lower in comparison to images acquired with full time protocols, and a less aggressive reduction in scan-time is therefore recommended.

## Background

Long acquisition times in routine imaging procedures, such as whole body bone scintigraphy, are a constant issue in the field of nuclear medicine. Shorter acquisition times allow for more patient examinations per imaging modality and also a decrease in discomfort of patients suffering from painful diagnoses such as bone metastases. Whole body bone scintigraphy is one of the most common nuclear medicine investigations worldwide [1], and the recent increase in complementary use of SPECT-CT imaging [2] points towards a need for reduced scan-times in the years to come. The obvious reason for not reducing the acquisition times is the associated decrease in signal-to-noise ratio (SNR). This effect could be compensated for by an increase in patient administered activity; however, this approach is not necessarily consistent with keeping the dose to the patient as low as reasonably achievable. Another way to address the increase in noise associated with shorter acquisition times is to apply different post-processing methods such as Pixon® image processing.

The Pixon method utilizes a noise-reducing post-processing algorithm that minimizes image noise in the processed image while preserving vital information from the raw data [3, 4]. The algorithm is based on the principle that the ideal image is represented by the lowest possible number of parameters that correctly represent the raw data image, i.e., redundant parameters impair the image quality by decreasing the SNR and should therefore be suppressed. It is well known that image noise can be repressed by applying a smoothing filter on the entire image. However, oversmoothing in regions of high detail could lead to loss of diagnostically relevant information. The Pixon-algorithm addresses this issue through the employment of adaptive noise reduction which assigns a smoothing kernel for each pixel based on the level of detail in the region of neighboring pixels. The smoothing kernels form an adaptive noise map that constrains smoothing in regions of high detail and allows more smoothing in large homogenous areas of the image.

Previous studies employing Pixon-algorithms on planar images have shown promising results with improved image quality in ^99m^Tc nuclear medicine images supporting shorter acquisition times or lower administered activities in conjunction with the Pixon-algorithm [3–6]. These studies have either applied the algorithm to existing clinical full scan-time images, to clinical images with Gaussian noise added to simulate shorter acquisition times or to phantom studies. Despite promising results, the Pixon-algorithm is not commonly employed in clinical practice [6], indicating the need for further studies employing images acquired with clinical protocols and reviewed by experienced clinical observers.

Thus, the aim of the present study was to evaluate a commercially available Pixon post-processing noise reduction algorithm applied on clinical ^99m^Tc-diphosphonate whole body bone scintigraphy images. More specifically, can a planar image acquired with half the acquisition time and post-processed with a Pixon-algorithm provide the reviewing physician with the same clinical information as a full scan-time non-processed image?

## Methods

### Image acquisition and processing

Twenty patients (16 male, 4 female) with a mean age of 72 years (range 58–84) scheduled for whole body bone scintigraphy were selected for the study which was approved by the Regional ethics research committee (Regionala etikprövningsnämnden i Stockholm; Karolinska Institutet, SE-171 77 Stockholm, Sweden). All patients were included on the criteria of confirmed or strongly suspected bone metastases. This was established from a prior radiographic/CT-scan or from the patient being examined as part of a treatment follow-up. Inclusion was made during a time period of approximately 9 months in which patients were consecutively selected for the study. Weights and heights were collected for all patients to investigate potential correlations between body mass index (BMI) and the results from the evaluation.

The patients were administered with 500 MBq ^99m^Tc-oxidronate (TechneScan™ HDP, Mallinckrodt Medical B. V., Petten, The Netherlands) and scanned on a Siemens Symbia T16 system approximately 3 h after injection. Each patient was first imaged using a standard clinical protocol with a table feed of 12 cm/min corresponding to approximately 1.5 million counts per anterior and posterior image, according to the EANM guideline recommendations [7]. Following the first image acquisition, each patient remained in the scanner and was subsequently imaged using a protocol with half the standard scan-time, corresponding to a table feed of 24 cm/min. Thus, a total of 40 images (20 patients with 2 images each) were acquired. Any variation in patient radiopharmaceutical uptake between the two scans was neglected due to the short-time interval between the two acquisitions in comparison to the total uptake time.

**Fig. 1:**
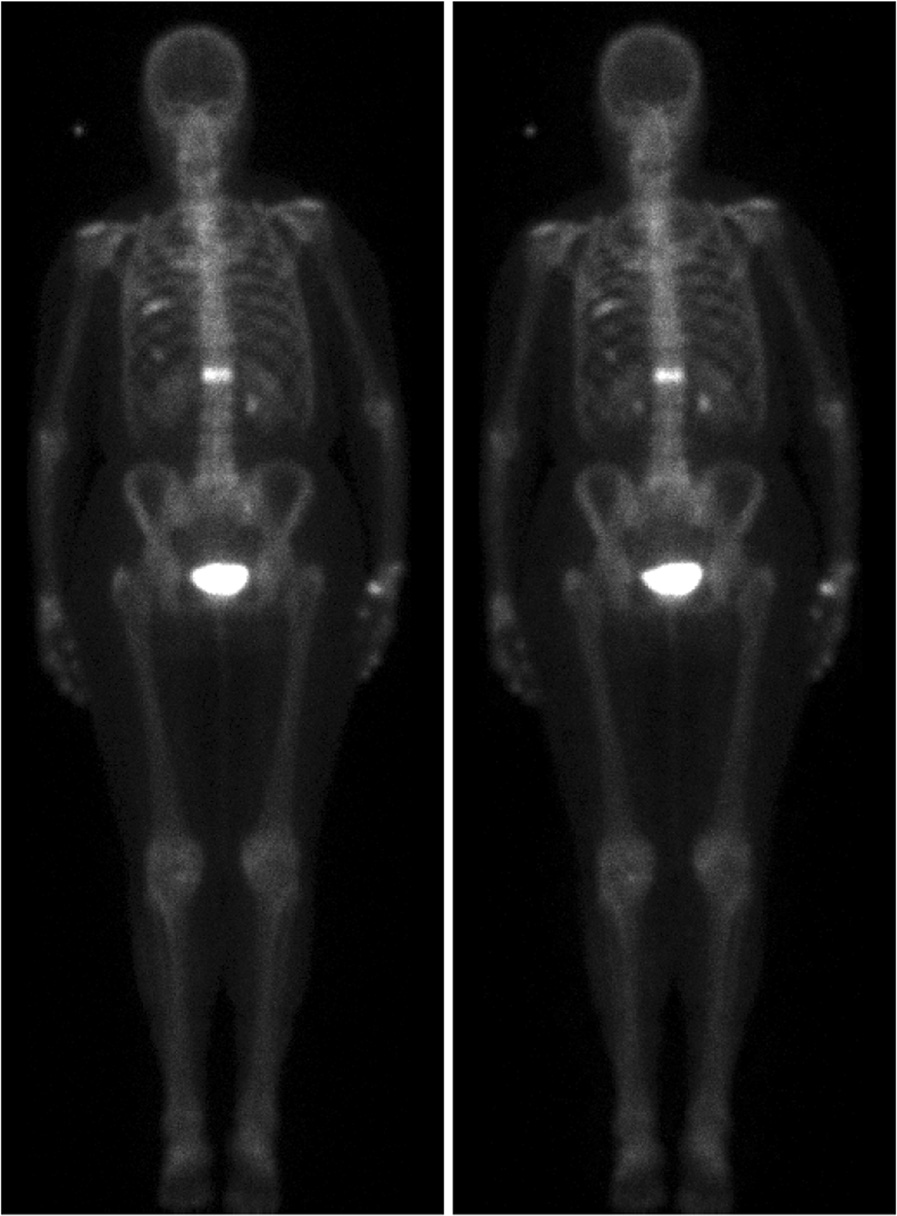
Images corresponding to a typical patient. *Left*: image acquired using the standard full-time protocol. *Right*: image acquired using the half-time protocol and processed with the Pixon-algorithm

All images were obtained using low energy high resolution collimators and a 256 × 1024 matrix. Half-time images were processed using Siemens Pixon Enhanced Planar Processing which is a commercially available software package optimized for whole body bone scintigraphy combining the original half-time image and a Pixon-processed image generating a final processed half-time image. All available software parameters were set to default values recommended by the vendor (α-parameter = 0.3, denoise parameter = 1.7, maximum kernel radius = 10, number of kernels = 12, maximum iterations = 20). Images acquired using the standard full time protocol and the processed half-time protocol corresponding to a typical patient is presented in Fig. 1.

### Evaluation

All images were visually evaluated by three experienced nuclear medicine physicians with several years of experience in interpreting bone scans, and all evaluations were performed individually. Images were anonymized with regard to both patient data and scan protocol. The evaluation consisted of two parts; a single evaluation of all images and a comparison study of all image pairs. In both evaluations, the observers scored the images according to a set scale and were also able to add comments.

The single evaluation was performed prior to the comparison study in order to minimize bias effects. All 40 images (20 processed half-time images and 20 standard images) were evaluated in randomized order. The image quality was graded as *poor*, *sufficient*, or *good* with regard to detectability of diagnostically relevant findings. Also, to evaluate if the performance of the algorithm was dependent on patient size, the results from the single evaluation were grouped into four intervals of patient BMI (<20, 20–25, 25–30, >30), and the image quality grading in each interval was determined.

In the comparison study, 20 randomized image pairs of one processed half-time image and one standard image per patient were evaluated against each other. The physicians were asked to rank the images in each image pair with regard to *diagnostically relevant difference in lesion detectability*, *occurrence of artifacts*, and *level of image noise*.

## Results

The results in Table 1 show that observer A and B rated all images as *sufficient* or *good* with regard to detectability of diagnostically relevant findings, whereas observer C rated four processed half-time images and one standard image as *poor*. The results of the single evaluation showed small differences between observer ratings as only 2 of the 40 images were inconsistently graded, i.e., the same image was ranked *good* by one observer and *poor* by another. The processed half-time images were predominately considered *sufficient* (65 %), whereas the majority of the standard images were graded as *good* (83 %). This was also seen for the patient presented in Fig. 1 where the rating of the standard image with regard to diagnostic detectability was *good* by observer A and B, and *sufficient* by observer C, while the processed half-time image was rated as s*ufficient* by all observers. Observer B found that three of the processed half-time images were noisy and on the verge of having poor image quality. The corresponding standard images were all rated as *good* by the same observer.

**Table 1:** Results from visual single evaluation with regard to detectability of diagnostically relevant findings of *n* = 40 images (20 images acquired with the standard protocol and 20 processed half-time images)

	Processed half-time images	Standard protocol images
Observer A		
Poor	0 (0 %)	0 (0 %)
Sufficient	17 (85 %)	1 (5 %)
Good	3 (15 %)	19 (95 %)
Observer B		
Poor	0 (0 %)	0 (0 %)
Sufficient	11 (55 %)	3 (15 %)
Good	9 (45 %)	17 (85 %)
Observer C		
Poor	4 (20 %)	1 (5 %)
Sufficient	11 (55 %)	6 (30 %)
Good	5 (25 %)	13 (65 %)
Combined		
Poor	4 (7 %)	1 (2 %)
Sufficient	39 (65 %)	9 (15 %)
Good	17 (28 %)	50 (83 %)

The results from the comparison study in Table 2 show that observer A and C mostly preferred the standard images and that observer B did not have any preference in any of the 20 image pairs with regard to lesion detectability. With regard to the level of image noise and artifacts, the standard images were rated superior in 65 and 23 % of the evaluations. The most common manifestation of image artifacts reported by the observers was represented by small regions of signal drop within soft tissues. These soft tissues were in some cases also reported to contain high levels of image noise.

**Table 2:** Results from visual comparison study with regard to lesion detectability, image noise, and artifacts of *n* = 20 image pairs (20 images acquired with the standard protocol and 20 processed half-time images)

	Processed half-time image preferred	No preference	Standard protocol image preferred
Observer A			
Lesion detectability	2 (10 %)	9 (45 %)	9 (45 %)
Image noise	0 (0 %)	3 (15 %)	17 (85 %)
Artifacts	0 (0 %)	14 (70 %)	6 (30 %)
Observer B			
Lesion detectability	0 (%)	20 (100 %)	0 (0 %)
Image noise	0 (%)	11 (55 %)	9 (45 %)
Artifacts	0 (%)	14 (70 %)	6 (30 %)
Observer C			
Lesion detectability	5 (25 %)	5 (25 %)	10 (50 %)
Image noise	3 (15 %)	4 (20 %)	13 (65 %)
Artifacts	1 (5 %)	17 (85 %)	2 (10 %)
Combined			
Lesion detectability	7 (12 %)	34 (57 %)	19 (32 %)
Image noise	3 (5 %)	18 (30 %)	39 (65 %)
Artifacts	1 (2 %)	45 (75 %)	14 (23 %)

The results from the BMI evaluation are presented in Table 3 where the ranking of all observers corresponding to the single evaluation with regard to detectability of diagnostically relevant findings of each BMI interval is presented. The results show that the standard images were predominantly rated as *good* in all BMI intervals except for BMI > 30 (50 %), whereas the majority of the processed half-time images were rated as *sufficient* in all BMI intervals.

**Table 3:** Results of single evaluation with regard to detectability of diagnostically relevant findings of all observers presented in patient BMI intervals. The numbers in the parenthesis correspond to the number of patients in each BMI interval

	<20 (1)	20–25 (8)	25–30 (9)	>30 (2)
Standard images				
Poor	0 %	4 %	0 %	0 %
Sufficient	0 %	21 %	15 %	50 %
Good	100 %	75 %	85 %	50 %
Processed half-time images				
Poor	0 %	8 %	4 %	17 %
Sufficient	67 %	67 %	54 %	83 %
Good	33 %	25 %	42 %	0 %

## Discussion

Previous studies have demonstrated that the Pixon-algorithm improves the image quality when applied on planar images acquired with full time acquisition protocols both with regard to an increase in SNR and improved observer sensitivity and specificity [3, 4]. Other studies have used the Pixon-algorithm on images retrospectively resampled to simulate a decrease in acquisition time and have demonstrated both negligible effects [6] and subjective improvement in image quality [5]. The present study focused on using the noise-reducing Pixon-algorithm on images acquired with a clinical protocol to compensate for the reduction in image counts associated with the transition from standard acquisition times to substantially decreased acquisition times. The purpose of this approach was to investigate how the change in image characteristics associated with the Pixon-algorithm applied on images actually acquired with half the standard acquisition time would affect clinical observers when compared to an existing full-time clinical protocol.

The performance of the Pixon Enhanced Planar Processing algorithm was indeed manifested, as the considerable reduction in image acquisition time by a factor of 0.5 and the consequent decrease in signal-to-noise ratio by a factor of 0.7 ($\sqrt{1/2}$) were expected to substantially lower the overall image quality. These large differences in image quality were however not seen when swiftly comparing the images as is seen in the similarity of the patient images presented in Fig. 1. Nevertheless, the results in Table 2 demonstrate that the observers ranked the standard images as superior with regard to image noise in 65 % of the evaluations. This implies that the effect of Pixon Enhanced Planar Processing on overall image quality does not fully cancel out the consequences of reducing the scan-time in half.

When assessing lesion detectability, the observers reported no preference between image types in 57 % of the patients as seen in Table 2, indicating that the processed half-time images provide sufficient information with regard to diagnostically relevant findings in a majority of the evaluated images. This claim is supported by the results from the single evaluation presented in Table 1, where 93 % of the processed half-time images were rated as *sufficient* or *good* by all observers. Thus, when applying the Pixon-algorithm on half scan-time images, the most important evaluation parameter, lesion detectability, appears to remain relatively unaltered.

Processed half-time images were prone to show a higher degree of image artifacts in comparison with the standard images as shown in Table 2. These artifacts were mostly observed in soft tissues indicating that the Pixon-algorithm may not be suitable for examinations where high-image quality in soft tissue is desirable.

Due to that the standard images were acquired prior to the half-time processed images, a potential bias effect originating from differences in background clearance between the two acquisitions was possible. As a consequence, the bladder activity was visually compared between both acquisitions for all patients. This comparison showed no difference in uptake between the acquisitions indicating that any bias effects due to differences in clearance rate could be ruled out.

A possible approach to improve the outcome when implementing the Pixon-algorithm could be to alter the default settings of the parameters used by the algorithm on a patient-to-patient basis. However, the results in Table 3 demonstrate that the processed half-time images were similarly rated independent on patient BMI. This indicates that the performance of the algorithm is unaffected by patient size and that the use of constant default parameter settings applied in the present study is reasonable. Nevertheless, altering the parameters could improve the image quality of the Pixon-processed images with regard to its overall performance regardless of patient anatomy.

The results in Table 2 demonstrate the difficulties associated with inter-observer variability when conducting subjective evaluation studies as the present one. Thus, observer A and C preferred the standard images in a much larger extent in comparison to observer B. The reason for this result could be bias effects originating from the experience of the observers reviewing standard images and consequently favoring these. All images were anonymized and the single evaluation was carried out prior to the comparison study to minimize bias effects, but as the observers learned to identify the half-time processed images, the possibility of such influence cannot be ruled out. Nevertheless, the results of the single evaluation presented in Table 1 shows that only observer C ranked four of the processed half-time images as *poor* supporting the conclusion that the majority of the Pixon-processed images provide sufficient diagnostic information with regard to lesion detectability.

## Conclusions

The findings of this study showed that implementing a Pixon-algorithm on whole body bone scintigraphy images acquired with half the acquisition time in overall provide sufficient clinical information with regard to detectability of diagnostically relevant findings. The performance of the algorithm proved equivalent regardless of patient size. To reduce the image noise and number of artifacts associated with the processed half-time images, a less aggressive reduction in scan-time is suggested for implementation of the Pixon-algorithm.
